# ”Sociobiome”: How do socioeconomic factors influence gut microbiota and enhance pathology susceptibility? - A mini-review

**DOI:** 10.3389/fgstr.2022.1020190

**Published:** 2022-11-07

**Authors:** José Guilherme Nobre, D. Alpuim Costa

**Affiliations:** ^1^ Faculdade de Medicina da Universidade de Lisboa, Lisbon, Portugal; ^2^ Instituto de Saúde Ambiental e Saúde Pública (ISAMB&SP), Lisbon, Portugal; ^3^ PTSurg – Portuguese Surgical Research Collaborative, Lisbon, Portugal; ^4^ Haematology and Oncology Department, CUF Oncologia, Lisbon, Portugal; ^5^ NOVA Medical School, Faculdade de Ciências Médicas, Lisbon, Portugal

**Keywords:** gut microbiome, health, disease, socioecomic status, sociobiome, gut microbiota

## Abstract

The gut microbiota is becoming well recognized as a key determinant of health and disease. As a result, several studies have focused on causality and the predictive/prognostic value of the microbiota in a wide range of diseases. However, it is of greater importance to understand what sparks changes in the microbiota and how these alterations contribute to an increased susceptibility to disease. A few studies have already demonstrated that the gut microbiota could be modified by lifestyle, consequently leading to pathology. What if socioeconomic factors can also impact the gut microbiota composition and, thus, increase the susceptibility to disease? Perhaps, this is one of the factors that may have contributed to the increased inequalities between people with higher and lower socioeconomic status in terms of health. In this review, we aimed to understand more about this topic and the real impact of the “sociobiome.” Furthermore, we proposed measures to mitigate the impact of these factors on the gut microbiota composition.

## Background

Eradication of poverty was listed as one of the main Millennium Development Goals (MDGs) to be tackled by the WHO, especially in low-income countries (1). Thus, it indicates that health is a key determinant for increasing the socioeconomic status (SES) and, hence, can influence an individual’s success throughout life. Therefore, achieving the best health odds at a young age is important.

The microbiota is intrinsically correlated with health and disease, making it promising to understand part of the pathophysiology, which, in turn, can help in the achievement of a healthier status, especially due to the therapeutic potential to modulate the composition of the microbiota.

The microbiota consists of a plethora of microorganisms, including bacteria, protozoa, archaea, viruses, and fungus, that inhabit mainly the intestines, as well as other sites of our organism, which establishes a symbiotic relationship with us. It is acquired at the moment of birth, either through vaginal or cesarean delivery, which presents as one of the first interferents of the microbiota composition, diversity, and disease susceptibility in the future (2). The establishment of a more mature, balanced, and diverse state of microbiota composition is obtained at the age of 4 years, which is divided into three main stages: 1) the developmental period (at 1 year old), where the child’s microbiota is influenced by breastfeeding, geographics, maternal and/or fetal diseases, and the use of antibiotics; 2) the transitional period (at 2 years old), where exposure to the environment, such as pets, siblings, other household related-acquaintance, and chronic pathologies, among others, increases and affects the microbiota; and 3) the stable period (at 4 years old), which will remain throughout life and can be slightly modified by lifestyle and diet (2, 3).

Moreover, it is important to understand the impact of ethnicity and geographic location. One interesting study performed in Indian tribes revealed that their microbiota was dominated by *Prevotella* spp., with just slight changes at the genus and species levels mainly due to different diet nuances. Additionally, a representative microbiota core was detected, similar to that of most world populations, with *Faecalibacterium*, *Eubacterium*, *Clostridium*, *Blautia*, *Ruminococcus*, and *Roseburia* (4). Furthermore, another study, reporting on some of the tribes included in the previous one, demonstrated that the microbiota composition and respective metabolomics are shaped by ethnicity (5).

On another side of the world, specifically South America, an Amerindian tribe without previous contact with westernized people was discovered to have the most diverse and functional microbiota ever documented, indicating that exposure to westernized culture affects our collective microbiota composition (6).

Before delving further into the effects of socioeconomic features on the dynamics of the microbiota, it is important to remember that xenobiotics, including exposure to medications and environmental toxins, are a major contributor to microbiota dysbiosis. After all, westernized populations may be more exposed to these xenobiotics, leading to innumerous pathologies, especially in populations with fewer resources (7–9).

**Figure 1:**
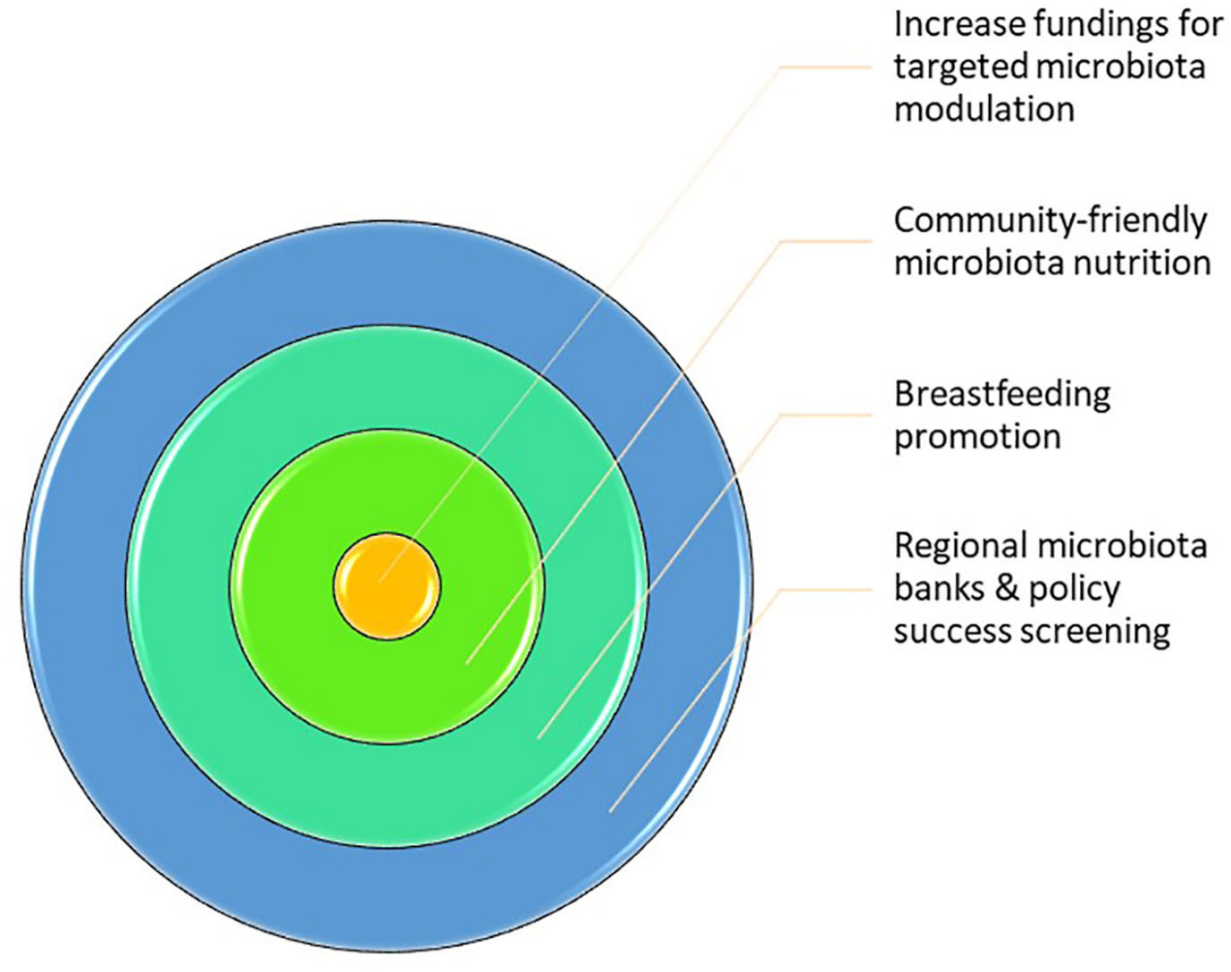
Sociobiome key points. Hereby, it is possible to get this conclusion the following conclusion: 1) Socioeconomic status (SES) has a higher contribution to microbiota composition than genetics; 2) SES is extremely important in the development of children that it also affects the adult microbiota; 3) Either individual or community SES is relevant for microbiota composition; therefore, community measures can be applied more easily and benefit even more individuals.

Furthermore, it appears that household exposure can be separated into household crowding and SES, especially at a young age. This may shape the individual microbiota, determining the susceptibility to disease and, hence, their chances of success in accomplishing higher SES. Therefore, the main aim of this article was to explore the importance of SES in the predisposition to pathology through its influence on the microbiota composition. The main findings are summed up in **Figure 1**.

## Socioeconomic status *vs.* inheritability

As previously described, the microbiota is acquired at the moment of birth by transference from mother to newborn. Hence, logically, the greatest contributor to microbiota composition would be genetically determined. There are even some taxa and species that are already depicted as highly heritable. The study by Gacesa et al. (10) in a Dutch population found that some bacteria, including *Proteobacteria*, *Akkermansia muciniphila*, Bacteroidaceae species, *Parabacteroides goldsteinii*, *Bacteroides coprocola*, *Bifidobacterium longum*, *Phascolarctobacterium*, and Clostridiales, are genetically transmissible. Other studies in a Canadian population and a cohort of UK twins reported similar findings (11, 12).

Nevertheless, most studies demonstrated that cohabitation and/or SES are more important in determining the composition of the microbiota than inheritability. The Dutch study (10) showed that the intestinal microbiota of family members living separately has a lower resemblance compared to household members, even if there was no significant relationship between them. However, some bacteria of inheritability signature still contribute to the microbiota composition, to a lower degree. Moreover, another UK twin study that isolated the genetic contribution demonstrated the greater magnitude of SES in the structure of the microbiota (13).

## Influence of socioeconomic status on a child’s microbiota composition that reflects in adulthood

The microbiota composition, maturity, and diversity are stabilized in the fourth year of life (2), which means that all exposure during the first years will have enormous significance on the health status and, indirectly, on the SES. Thus, the Dutch study (10) demonstrated that the childhood milieu is reflected in the adult microbiota configuration through a comparison between rural and urban environments. It was described that children residing in urban environments showed lower abundance of *Bacteroides*, *Alistipes*, and *Bilophila* compared to those in rural backgrounds. Conversely, the microbiota of children in rural areas was enriched in *Prevotella copri*, *Faecalibacterium prausnitzii*, *Rothia muciliginosa*, *Bifidobacterium* spp., and *Mitsuokella* (10). All the previous bacteria from rural microbial signatures have anti-inflammatory characteristics that may enhance the resilience of the microbiota and decrease the susceptibility to disease. These findings are described in **Table 1**.

**Table 1:** Discriminate SES.

Geography	High SES	Low SES	Conclusions	References
**Netherlands (Europe)**	↓ *Bacteroides* ↓ *Alistipes* ↓ *Biophile*	↑ *P. copri* ↑ *F. prausnitzii* ↑ *R. muciliginosa* ↑ *Bifidobacterium spp.* ↑ *Mitsuokella*	Low SES children seems to have more resilient microbiota and develop less diseases	(10)
**Israel (Asia)**	↓ *A. Onderdonkii* ↓ *B. uniformis* ↓ *P. stercorea* ↓ *Phascolarctobacterium* ↓ *A. Putrensis* ↑ Secondary Bile Acids biosynthesis ↑ Glutamate and Glutamine metabolism ↑ Biotin metabolism	↑ *P. copri* ↑ *A. Putredinis* ↑ *E. biforme* ↑ *Dialister* ↑ *F. prausnitzii* ↑ *Bifidobacterium spp.* ↑ *Oscillospira* ↑ *Ruminococcus* ↑ *Sutterella*	Lower SES children present higher BMIZ, are more prone to obesity and had less bacterial diversity, depending in the quantity of fiber present in their diets	(14, 15)
**Mexico (South America)**	↑ *Saccharibacteri*	↑ *Dinococcus-Thermus* ↑ *Chloroflexi* ↑ *Elusimicrobia* ↑ *Acidobacteria* ↑ *Fibrobacter*	High SES children had increased amounts of sugar decomposing-bacteria, indicating an enhanced propensity to obesity	(16)

Furthermore, the study of Lapidot et al. (14) in Israel has shown that household crowding and SES are major contributors to the bacterial composition of young children, mainly through increasing the alpha diversity and phylogenetic variety. A lower SES was associated with a wider taxonomic range, consisting of *P. copri*, *Alistipes putredinis*, *Eubacterium biforme*, *Dialister*, *F. prausnitzii*, *Bifidobacterium*, *Oscillospira*, *Ruminococcus*, and *Sutterella*, which, in turn, are astonishingly similar to the microbiota signatures of those in rural communities (10, 14). This might be explained by the fact that individuals from villages in Israel with lower SES have decreased monthly wages, lower education levels, and are less exposed to Westernized diets, accompanied by augmented consumption of the Mediterranean diet. Moreover, household crowding showed differences in the abundance of *Alistipes onderdonkii*, *Bacteroides uniformis*, *Prevotella stercorea*, *Phascolarctobacterium*, and *A. putredinis*, with those having lower SES presenting a dominant taxon of *B. uniformis*, while a higher SES was mainly composed of *P. stercorea* and *Phascolarctobacterium* (14). The metabolic pathways were also different, with children in higher SES households showing overdeveloped secondary bile acid biosynthesis, which is important for its regulatory effect on inflammation and microbial composition (17); increased glutamate and glutamine metabolism, crucial to maintaining the intestinal barrier integrity (18, 19); and, finally, enhanced biotin metabolism, which is responsible for the metabolism of glucose, amino acids, and fatty acids (14, 20)..

Moreover, the same authors conducted a more recent study where it was observed that a lower SES showed not only significant microbiota alterations but also increased body mass index *Z*-score (BMIZ) in preadolescents (15). Children in reduced SES households demonstrated a higher prevalence of obesity, complemented with reduced bacterial diversity due to their main diet comprising higher quantities of dietary fat without increased consumption of fibers. Therefore, the microbiota of children with lower SES is enriched in *Prevotella*, *Adlercreutzia*, *Alistipes*, and *Dorea*, which have been correlated with obesity (21) and diabetes mellitus (15, 22). These findings are displayed in **Table 1**.

Furthermore, a study performed in Mexico comparing the different microbiota compositions of children in westernized (higher SES) and non-westernized (lower SES) settings reported that non-westernized children had unique phyla of bacteria, namely, Deinococcus-Thermus, Chloroflexota, Elusimicrobiota, Acidobacteriota, and Fibrobacterota, more related to a vegetable-based diet. In contrast, westernized children had diminished diversity and a more representative phylum of Saccharibacteria, one of the main functions of which is the decomposition of sugar molecules. To sum up, since non-westernized children are less exposed to sugar-containing foods and eat a more diverse range of vegetables, they appear to have a more resilient microbiota that is more efficient in harvesting the energy from fibers (16).

Nevertheless, it is important to highlight the role of *Prevotella*, present in both studies, since it is still the bacterium that is vastly abundant in the human intestine. However, this genus has already been correlated with positive and negative outcomes in health. On the one hand, *Prevotella* has been implicated in glucose intolerance and insulin resistance (23). On the other hand, when a diet rich in fiber is consumed, *Prevotella* improves glucose and insulin tolerance, which points to the fact that its benefits or risks are diet-induced (24).

## Socioeconomic status: Individual versus community

The composition of the microbiota is influenced by individual lifestyle, namely, diet, physical exercise, and individual SES, but is also highly dependent on neighborhood SES, which contributes to greenspace area, exposure to pollution and toxicants, stress, and the type of diet consumed, such as ultra-processed food (10, 25).

The study by Miller et al. (25) evaluated the influence of neighborhood SES on the microbiota composition in the mucosal and luminal locations of the sigmoid colon. It was noted that the alpha diversity was diminished in those in low-SES communities, which, in turn, showed higher rates of diabetes (26), cardiovascular diseases (27), asthma (28), and mortality. Moreover, an enhanced prevalence of *Bacteroides*, with a lower abundance of *Prevotella*, was reported in the microbiota of individuals belonging to higher-SES neighborhoods, probably due to better diets with increased consumption of animal products (25).

The alpha diversity, which reflects the evenness and richness of the microbiota, is a significant indicator of microbiota resilience (3). Hence, individuals with decreased alpha diversity, for example those belonging to lower-SES neighborhoods, showed less resilience, which means that they are more prone to pathologies (29).

Regarding individual SES, one study pointed out that measures of individual SES, particularly an individual’s monthly wage, is a determinant of alpha diversity (13). It was demonstrated that a higher individual SES correlated with an enhanced alpha diversity, with an increased abundance of *Bacteroides* and *Prevotella*, which was in contradiction with the results of the study of Miller et al. (25), which presented a reduced abundance of *Prevotella* (13).

## Sociobiome: What does the future hold?

Sociobiome can be defined as the microbiota composition of a geographic region or neighborhood as a result of exposure to similar socioeconomic factors, which determine an environment with analogous characteristics that shape the individual microbiota into great resemblance. Therefore, this sociobiome can be used to increase the success of health policies more personalized to a specific region instead of broad interventions across a territory full of diverse realities and dissimilar issues.

For instance, since the microbiota appears to interact with the development of the central nervous system, as well as the regulation of individual behavior (30), there is a possibility that not only does the SES affect a person’s microbiota but also, in a reverse mode, that the microbiota composition shapes the behavior of an individual in such a way that it regulates the capacity to influence SES and to acquire habitation in specific neighborhoods (25). With this being said, it opens the possibility of modifying health disparities due to SES since there are interventions, especially those aimed at the youth, that can be fashioned to shape the microbiota of those with lower SES in order to ameliorate present and future health problems.

**Figure 2:**
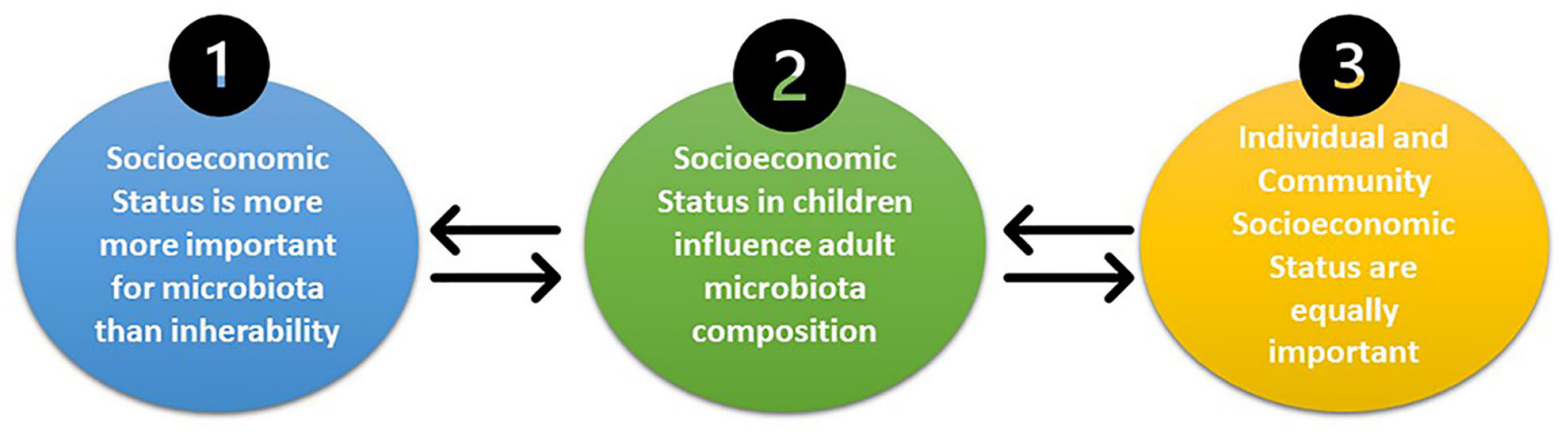
Future sociobiome interventions that might increase the quality of the community microbiota. The main interventions suggested in this manuscript are: 1) increased funding for targeted microbiota interventions; 2) community-friendly microbiota nutrition; 3) breastfeeding promotion; and 4) regional microbiota banks and policy success screening.

Hereby, we suggest some interventions that can decrease the chasm between low and high SES and equalize the health status, as reflected in **Figure 2**.

1. *Increase fundings for targeted microbiota modulation* (31): It is necessary to develop research on health disparities based on microbiota differences in order to obtain “antidotes” that can be used to modulate the microbiota through increasing the alpha diversity, which, in turn, will enhance the microbiota resilience and ameliorate the health status. Here, personalized therapies for microbiota modulation, such as combinations of probiotics, prebiotics, symbiotics, and antibiotics, should be developed, as well as the possibility of fecal material transplant (FMT). Furthermore, this intervention should be directed at children since it appears to have a “founder effect,” with adult SES indicating a cumulative acquaintance throughout life (13).
2. *Community microbiota-friendly nutrition*: The impact of nutrition on the microbiota composition is well recognized. Hence, it would be important for the population, especially children, to have access to the best food available instead of high-fat, high-carbohydrate, and low-fiber diets. As previously shown, *Prevotella*, which has an important impact on health disparities, is highly dependent on diet, namely, fiber; hence, it is necessary to increase the fiber intake, with the aim of enhancing the beneficial effects of *Prevotella* (31). Therefore, food banks and food supplement programs should be available for children in order for them to benefit from the best diet possible. Additionally, to promote healthier nutrition, high-fiber and fresh food should have reduced taxes and/or budget supplements for low-SES families, contrarily to high-fat products that should have increased taxes.
3. *Breastfeeding promotion*: One of the first major modulators of the microbiota in youngsters is breastfeeding. However, breastfeeding is often difficult to maintain in low-SES families due to the need to work to support their families. Thus, workplaces should allow breastfeeding periods and/or receive statal support to facilitate breastfeeding (31).
4. *Regional microbiota banks and policy success screening*: Since microbiota sequencing is becoming more accessible, individual microbiota should be examined in a standardized periodicity for the optimization of health policies and evaluation of their success, along with the possibility of early detection of disease and modulation of the microbiota. Furthermore, microbiota samples could be stored under optimal conditions and, in the not-so-far future, could be transplanted in an autologous manner to restore innate microbiota homeostasis when dysbiosis is detected.

## Conclusion

The microbiota has a huge impact on health and disease; subsequently, factors that can shape its composition, such as SES, have outstanding significance on the health status of an individual. Therefore, it is possible to understand that the sociobiome influences health disparities and can be targeted to reduce these inequalities. Moreover, the SES should be considered in microbiota research since it can be a crucial confounding variable that can influence the interpretation of the study outcomes.

SES appears to have a higher impact than heritability on the microbiota composition. Therefore, childhood interventions on the microbiota can increase the chances of an individual’s success throughout life, along with ameliorating the country’s productivity since there would be a reduction in the burden of disease. Furthermore, the sociobiome could lead to better screening of pathologies, accompanied by an enhancement in efficiency through tailored health policies specifically designed for certain neighborhoods.

To sum up, investing in personalized microbiota interventions in early life, especially in low-SES neighborhoods, could induce a win–win situation, where health disparities are attenuated alongside an increased productivity overall.

## Author contributions

The present manuscript is the result of the original work by the authors. JGN and DAC: Conception and design. JGN: Writing. DAC: Revision of the manuscript. Both authors contributed to the article and approved the submitted version. All authors contributed to the article and approved the submitted version.

## Conflict of interest

The authors declare that the research was conducted in the absence of any commercial or financial relationships that could be construed as a potential conflict of interest.

## Publisher’s note

All claims expressed in this article are solely those of the authors and do not necessarily represent those of their affiliated organizations, or those of the publisher, the editors and the reviewers. Any product that may be evaluated in this article, or claim that may be made by its manufacturer, is not guaranteed or endorsed by the publisher.
